# Spiritual Healing

**Published:** 1924-02

**Authors:** 


THE
### HOSPITAL and HEALTH
REVIEW

### SPIRITUAL HEALING.

"SPIRITUAL healing must not exclude medical means or methods," and "no sick person must look to the clergyman to do what it is the physician's or surgeon's duty to do." These two sentences sum up one of the most valuable conclusions arrived at by the Committee on "The Ministry of Healing," which has just been published by the S.P.C.K. at the inconsiderable price of sixpence. The Committee was appointed in accordance with a resolution of the Lambeth Conference of 1920, and, in addition to many learned theologians, it included half-a-dozen distinguished doctors — Sir Clifford Allbutt, Sir Robert Armstrong-Jones, Dr. William Brown, Dr. Hadfield, Dr. Jane Walker, and the late Dr. W. H. Rivers. This large infusion of professors of medical science confers upon the report a practical and intrinsic importance which it might have lacked had its conclusions been merely those of religious idealists anxious to believe, without too critical an inquiry, in the survival of those powers of healing which our Lord conferred upon the Church, using that ambiguous word in the sense of "the blessed company of all faithful people." Still greater importance attaches to the fact that the report is unanimous, with easily understood reservations by Sir Clifford Allbutt and Sir Robert Armstrong-Jones regarding the recommendation of the use of unction. That so considerable a company of healers of body and soul should be in agreement upon a subject of such grave moment, a subject, too, which has suffered much from extravagant claims and from a great deal of downright imposture, must needs compel serious public attention. It raises the whole question of the influence of mind upon body, and of the means by which the best remedial use may be made of that influence, to a distinctly higher plane. The Church of England has not been entirely blameless in giving tacit encouragement to, or at least in refraining from uttering words of serious warning against, certain campaigns of "spiritual healing" for which utterly extravagant claims of instantaneous cures have been made. No careful student of the subject would have the hardihood to declare that such cures never take place under certain conditions ; still less would he pronounce them to be impossible. But such cases are sufficiently rare to justify healthy scepticism.

The report recognises that there is a clearly marked line of demarcation between the functions of the healer of the body and those of the healer of the soul, while admitting that they may well, upon occasion, be partners in the process of restoration. There are physical disorders which have their root in spiritual or mental disturbance, and conversely ; and it is a fact of religious experience that the mere following, in faith of certain Sacramental ordinances sometimes has an ameliorative effect upon minor bodily troubles which depend upon psychological conditions. This much being ascertained, we need to take quite a short step to the probability that spiritual influences may exercise a large, and possibly a commanding, effect upon physical conditions more serious and more deeply rooted. The Committee, reasonably enough, lays stress upon the work of faith in securing restoration to health even by the primitive means of physic. The wonders which bottles of medicine are supposed to work are vastly helped by faith in the virtues of what may be little more mysterious than coloured water. Anxiety, depression, poverty, will produce physical results which it needs no argument to show may be at least as readily cured by spiritual suggestion as by directly medical treatment. These things are truisms, but they are far removed from the ignorant and absurd presumption that grave bodily disease or disability may almost, as a matter of course, be healed or removed merely by psychological suggestion, or the laying on of the hands of some professional " healer," even when accompanied by faith and prayer on the part of the sufferer.

Not the least valuable passages of the report, indeed, are those in which the Committee utter a warning against the misuse of psycho-analysis. When analyses of that character are undertaken by those who lack wide medical experience great evils may result, since " in unskilled hands forces may be liberated in the patient's mind that the amateur healer is incapable of controlling." For the matter of that there are degrees of amateurishness, and at present even the most skilled are not altogether free from the taint. The pontifical airs and cocksure language of some of these professors of the vague and the unascertained simply cause the " plain man " to shrug his shoulders and pass on with a sneer. In this matter, so far, we are all empirics, and some of the best-known exponents are arch-empirics. In some respects, however, the report is, almost necessarily, inconclusive—it is not just yet that the subject will lend itself to clear-cut conclusions, or anything in the nature of codification. And we must bear well in mind the admission of the Committee that they have found no case of spiritual healing which could not be paralleled by similar cures wrought by psychotherapy without religion, or by " cases of spontaneous healing which often occur in the gravest cases in ordinary medical practice." It is instances of candour such as this which help to invest the report with that atmosphere of commonsense which is never more necessary than when we are dealing with imperfectly understood psychological forces. The last infirmity which mankind will shed is credulity, and, taken all round, the report is a useful antidote to credulity. It does not go as far as certain schools of religious thought might desire, but it does much to put a subject of high importance in a light which enables it to be discussed in the right spirit. Medicine will certainly have no quarrel with it. " The physician," says the Committee, " is conscious all the time that he is working with a mysterious partner which we call the *vis medicatrix naturæ*." If by anything in the nature of spiritual analysis of the underlying causes of bodily trouble, whether that analysis be conducted by the doctor or the priest, we can help this tendency of the system to recover its equilibrium, it is clearly our duty to make use of it to the fullest extent. Meanwhile we must needs continue, but in the spirit of real humility, our studies not only of the body, but of that elusive and formless entity which we call the soul—an entity which, instinct suggests, is subject to as many ills as the flesh, with the one amazing exception of death.

